# Elucidating the Role of Ion Suppression in Secondary Electrospray Ionization

**DOI:** 10.1021/jasms.3c00219

**Published:** 2023-10-16

**Authors:** Cedric Wüthrich, Stamatios Giannoukos, Renato Zenobi

**Affiliations:** † Department of Chemistry and Applied Biosciences, ETH Zürich, 8093 Zürich, Switzerland

## Abstract

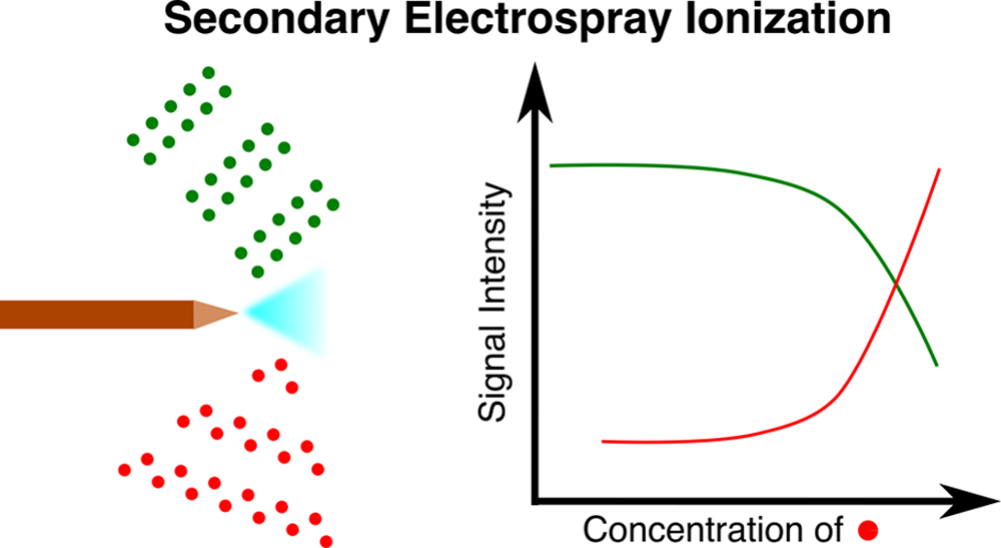

Ion suppression is a known matrix effect in electrospray ionization (ESI), ambient pressure chemical ionization (APCI), and desorption electrospray ionization (DESI), but its characterization in secondary electrospray ionization (SESI) is lacking. A thorough understanding of this effect is crucial for quantitative applications of SESI, such as breath analysis. In this study, gas standards were generated by using an evaporation-based system to assess the susceptibility and suppression potential of acetone, deuterated acetone, deuterated acetic acid, and pyridine. Gas-phase effects were found to dominate ion suppression, with pyridine exhibiting the most significant suppressive effect, which is potentially linked to its gas-phase basicity. The impact of increased acetone levels on the volatiles from exhaled breath condensate was also examined. In humid conditions, a noticeable decrease in intensity of approximately 30% was observed for several features at an acetone concentration of 1 ppm. Considering that this concentration is expected for breath analysis, it becomes crucial to account for this effect when SESI is utilized to quantitatively determine specific compounds.

## 1 Introduction

Secondary electrospray ionization (SESI) is a highly sensitive and soft ambient ionization method. It is coupled with high-resolution mass spectrometry (HRMS) and can be used to detect and monitor a wide range of volatile and semivolatile organic compounds.^1−5^ Although SESI offers many advantages (e.g., a higher sensitivity for polar and heavier compounds)^6^ compared to methods like selected-ion flow tube (SIFT) or proton-transfer reaction (PTR) mass spectrometry, it is still only semiquantitative. Quantification is possible only when calibration curves for specific metabolites can be obtained, which is time-consuming. Analyte concentrations are accessible with SIFT and PTR-MS systems because the ionization mechanism and many reaction rates are known.^7^ In contrast, the exact ionization mechanism of SESI is still a subject of debate,^8−10^ and thus, quantification must be performed with reference standards. External calibration would be desirable for ease of use, but matrix effects should be characterized beforehand.

Ion suppression is a matrix effect and a thoroughly documented phenomenon for electrospray ionization mass spectrometry (ESI-MS) coupled with liquid chromatography (LC). It describes the reduction of ionization efficiency through matrix compounds with a subsequent negative effect on sensitivity.^11−17^ Ion suppression has also been reported for APCI (ambient pressure chemical ionization), although with a diminished effect when compared to ESI.^14,15,18^

For flow-injection methods such as online SESI, the potential for suppression might be higher than for LC-ESI methods, as matrix compounds are not removed. For instance, samples with a high salt content require pretreatment to reduce suppression.^19^ A lower suppression effect has been reported for desorption electrospray ionization (DESI) and related ambient techniques, which do not require such pretreatment, compared to flow-injection ESI.^20−26^ A primary reason for these findings was the sampling separation from electrospray formation.

Some of these findings could apply to SESI, especially in the ambient, online analysis of breath. Other indications of how ion suppression could be affecting SESI have been reported by King et al.^11^ They investigated the ion suppression mechanisms in ESI and APCI. A conclusion that was reached was that gas-phase processes do not dominate ion suppression; rather, the presence of nonvolatile analytes prevents smaller droplets from forming and causes precipitation. One of their experiments closely resembled the SESI analysis of exhaled breath: two electrosprays were assembled in parallel, and the intermediate space between them was sampled. It was found that ion suppression in one spray did not affect the ionization performance of the other. According to these findings, one would assume that SESI would not suffer from ion suppression. However, indications to the contrary have been reported by Spesyvyi et al.^27^ One mechanistic hypothesis of SESI involves ligand switching, wherein the transfer of charges is mediated by the exchange of water molecules in a small, charged water cluster.^28^ Ion suppression would occur in this mechanism via a gas-phase process, where an abundant molecule replaces low-abundance molecules from these clusters. Acetone, a major component of breath, is therefore expected to be a prime candidate for causing suppression in online SESI breath analysis due to its high concentration levels (500 parts per billion (ppb) to 1 part per million (ppm))^29^ in exhaled breath.

Given the lack of thorough investigations into ion suppression in SESI, particularly in online breath measurements, these effects were investigated in detail. In this study, a gas generation system^30^ was used to examine the effect of variable compound concentrations on signal intensities. Acetone is of particular interest due to its high concentration in exhaled breath and its detection in positive ion mode, which is commonly used in clinical applications. This study provides clear evidence for ion suppression in SESI-MS, implicating gas-phase acid–base chemistry as a crucial factor.^6,31^ The quantitative nature of this work reveals the gas-phase concentrations at which signals are affected. This facilitates the interpretation of SESI-MS results and suggests ways to improve quantification.^31,32^

## 2 Methods

### 2.1 Reagents

Optima LC-MS grade water (Fisher Scientific) was used for all aqueous solutions. Formic acid (≥99.99% purity, Sigma-Aldrich) was dissolved in water with a volume concentration of 0.1% for the sprayed electrolyte solution. Acetone (≥99.5% purity) and D_6_-acetone (≥99.96% purity) were purchased from Sigma-Aldrich. D_3_-acetic acid (D_3_-AcOH, ≥99.5% purity) was purchased from Acros Organics, and pyridine (≥99% purity) was purchased from VWR Chemicals.

### 2.2 Experimental Setup

**Figure 1:**
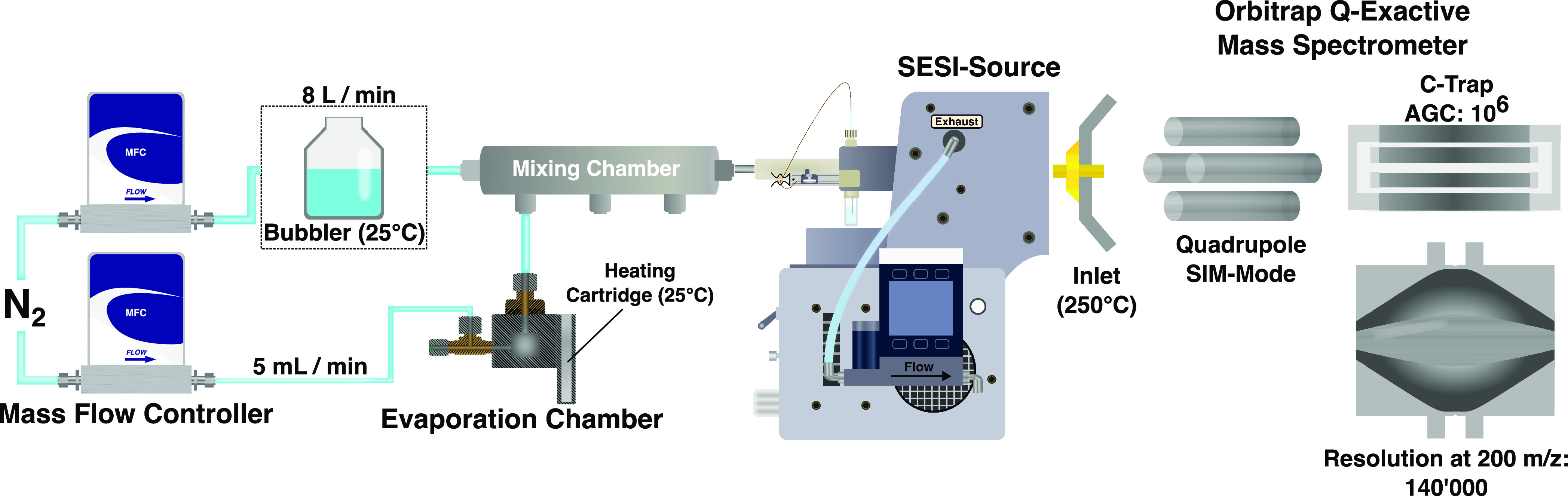
Instrumental configuration for the experiments presented here. Depending on the experiment, one, two, or three evaporation chambers were connected to the mixing chamber. Separate controllers regulated the flows passing through the evaporation and mixing chambers (dilution flow). A bubbler could be used to add moisture to the dilution gas. The mixing chamber outlet was connected to a commercial SESI source, which was attached to a Q Exactive Plus Orbitrap mass spectrometer. Reprinted in part with permission from ref (30). Copyright 2018 Elsevier, CC-By NC 3.0.

A previously described system that is based on evaporation chambers generated the individual gas standards.^30^ The principle of this system relies on Henry’s law, which describes the equilibrium between a gas and a solution at a known concentration. For the experiments described here, the following dimensionless Henry constants at room temperature were utilized: 0.67 for D_6_-acetone, 99.15 for D_3_-AcOH, and 2.73 for pyridine.^32^ For the deuterated compounds, the same Henry constants as those for the nondeuterated analogues were assumed. The setup that was employed is shown in Figure 1.

A dilution gas with a flow of 8 L min^–1^ was applied for all experiments conducted under either dry or humid conditions. Relative humidities of 0% and 95% at the mixing chamber outlet were obtained by either excluding or including a bubbler in the path of the dilution gas flow, respectively. The mixing chamber was kept at 60 °C to prevent condensation. An aqueous solution of a compound of interest was prepared and injected into one evaporation chamber connected to the mixing chamber to generate a gaseous standard. A 5 mL min^–1^ nitrogen flow passing through the evaporation chamber pushed the headspace established over the solution into the mixing chamber, thus diluting it by a factor of 1600. The ideal gas law was used for all analytes to calculate the final concentrations in ppm.

The analysis of the different concentration runs was conducted with a SUPER SESI ion source (Fossil Ion Tech) controlled by a flow meter (EXHALION, Fossil Ion Tech) coupled to a Q Exactive Plus Orbitrap mass spectrometer (Thermo Fisher Scientific). The gas standard generation system was attached to the sample inlet of the ion source via a home-built adapter connected to the flow meter. The sampling inlet was kept at 130 °C to prevent condensation, and the ionization chamber was kept at 90 °C. The electrolyte that was used for establishing the spray consisted of 0.1% (v/v) formic acid and the analyte at various concentrations. The electrolyte vial was subjected to an overpressure of 0.8 bar to generate the electrospray. The solution passed through a nanoelectrospray capillary (inner diameter of 20 μm, outer diameter of 365 μm, Fossil Ion Tech). Spray formation was aided by a sheath gas pressure of 15 psi and an auxiliary gas flow of 2 a.u. (arbitrary unit); the spray voltage was set to +3.5 kV. The inlet capillary of the mass spectrometer was set to 250 °C. The acquisition parameters of the Orbitrap were set as follows: AGC (automatic gain control) was 10^6^, and inject time was 500 ms with a resolution of 140 000 at 200 *m*/*z*.

**Figure 2:**
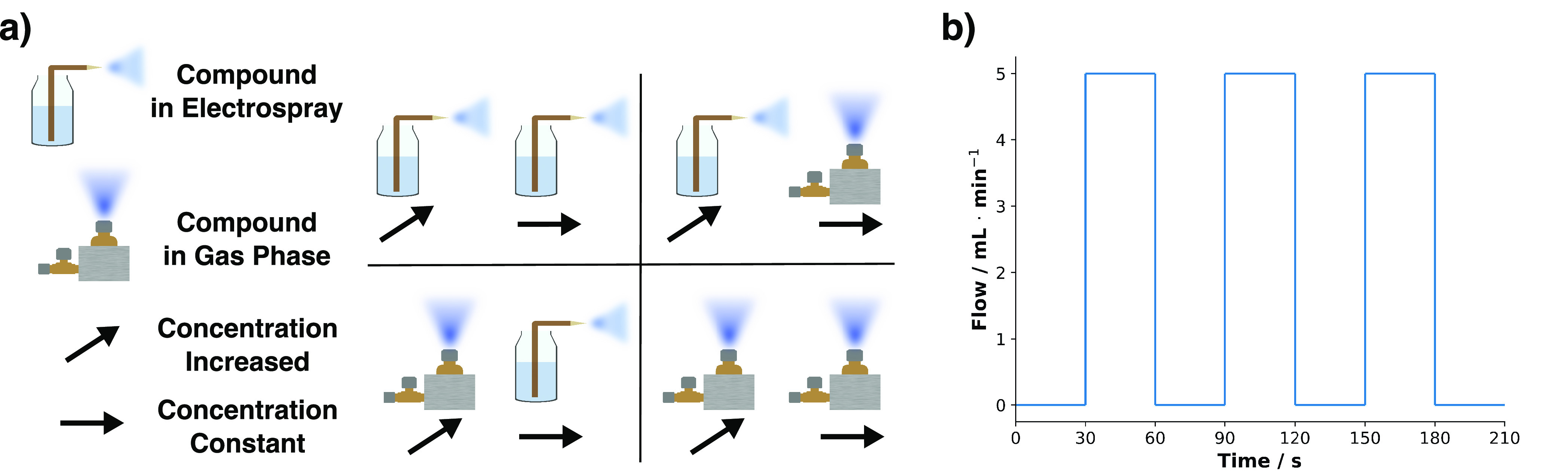
(a) Experimental design for investigating the effects of increasing the concentration of a compound in either the electrospray or the gas phase while keeping a competing compound constant in the other phase. The crossover experiments allowed for a classification of the ion suppression effect as occurring in either the liquid or the gas phase. These experiments were conducted with increasing acetone levels against D_6_-acetone, increasing D_6_-acetone levels against D_3_-AcOH, and the reverse of the latter. (a) The flow pattern through the evaporation chamber that generated the gas standard. The flow through the chamber was turned on and off in pulses: it was on for 30 s and then off for the next 30 s. This pulsating flow allowed the compounds in the system to be washed out through a large dilution gas flow through the mixing chamber. The signal intensity was determined through three replicates.

To establish the ion suppression effect of a selected compound in either the electrospray or the gas phase against a competing compound kept at a specific concentration, a first round of experiments was conducted, as depicted in Figure 2a.

This crossover experiment was conducted once with acetone and D_6_-acetone, where the acetone concentration was increased and the D_6_-acetone levels were kept constant. The same reaction thermodynamics expected from isotopic molecules allowed for use to gauge the effect of concentration without thermochemistry coming into play. Increases were done in half-logarithmic steps to cover a broad range of concentrations, with final concentration levels of the increased compound reaching a level that was 1000× higher than the initial concentration. Gaseous analytes were produced via the flow program illustrated in Figure 2b at a temperature of 25 °C. A new concentration required either the exchange of the electrospray solution or the injection (∼10 μL) of a different liquid stock solution into the evaporation chamber. Two additional rounds of this type of experiment were conducted with D_6_-acetone and D_3_-AcOH, with both compounds being increased once and kept constant once. The initial concentrations in the electrospray and gas phases are depicted in Table 1, which also correspond to the concentrations that were set constant in part of the experiments. The increases in concentration were conducted in steps of half and full orders of magnitude.

**Table 1:** Lowest Concentrations of the Selected Compounds Tested during the Crossover Experiments in Either the Electrospray or the Gas Phase^a^

compound	lowest concentration in the electrospray (M)	lowest concentration of the gas phase (ppm)
acetone	1.1 × 10^–7^	1.1 × 10^–2^
D_6_-acetone	1.1 × 10^–7^	1.1 × 10^–2^
D_3_-AcOH	1.4 × 10^–7^	7.0 × 10^–4^

a The same concentration was set if the concentration of the compound was kept constant. Increases of the compound concentration were done in half-logarithmic steps.

To minimize potential space charge effects, the mass spectrometer was operated in selected ion monitoring mode with a window from 59 to 66 *m*/*z* for acetone and D_6_-acetone and from 64 to 66 *m*/*z* for D_6_-acetone and D_3_-AcOH to cover the *m*/*z* of the expected [M + H]^+^ ions.

In addition to the crossover experiments, which were completed under both humid and dry conditions, an additional set of gas-phase experiments was conducted in the same manner and with the same flow program with pyridine also present (initial gas-phase concentration of 2.7 × 10^–4^ ppm). In this three-component experimental system, the D_3_-AcOH and pyridine concentrations were kept constant and measured individually against increasing concentration levels of D_6_-acetone. At a later stage, the concentration levels of D_3_-AcOH and pyridine were increased against a constant D_6_-acetone concentration. In addition to these binary experiments, all three compounds were combined in dry and humid conditions, where one concentration was increased and the other two were kept constant. For these and the following gas-phase experiments, the SIM mode window with a width of 1 *m*/*z* was cycled through the *m*/*z* area for each expected [M + H]^+^ ion (*m*/*z* of window center: 59.1 for acetone, 65.1 for D_6_-acetone, 64.1 for D_3_-AcOH, and 80.1 for pyridine) to increase sensitivity and minimize the space charge effects.

To simulate the conditions of online breath analysis by SESI-MS and the effects ion suppression could have, breath condensate was measured against increasing D_6_-acetone concentration levels. For this purpose, one subject’s exhalations were condensed during 5 min at −77 °C (dry ice/isopropanol) in a cold trap and kept frozen over dry ice. Part of the exhaled breath condensate was thawed and injected (∼10 μL) into the evaporation chamber. The same flow program that was previously described was used with increasing D_6_-acetone levels starting from the same gas-phase concentration shown in Table 1 and with the same evaporation chamber temperature of 25 °C. Mass spectra were acquired through a previously reported spectral stitching technique to improve sensitivity.^33^

### 2.3 Data Processing and Analysis

The obtained RAW files were converted to the mzML format^34^ via ProteoWizard and subsequently processed and plotted with a custom-written Python script (v3.9) and the Matplotlib label lines library.^35^ This script calculated an average mass spectrum of all measurements and performed peak picking with a height filter at 10^4^ a.u. The corresponding peak widths were determined to be 90% of the peak height. The corresponding time traces were calculated by the integration of each individual peak in each measurement. In each measurement, the time traces and the three individual pulses of the evaporation chamber were determined through change point detection with the l2-norm implemented in the ruptures Python package.^36^ The last 10 scans in each pulse were averaged to yield the signal intensity of the analytes of interest with the corresponding standard error.

## 3 Results and Discussion

### 3.1 Role of Electrospray Solution and Gas-Phase Composition in Ion Suppression

To understand the degree of ion suppression in SESI, the first question to be addressed was whether the analytes in the gaseous sample were responsible for signal suppression, as proposed by Dryahina et al.^10^ Alternatively, ion suppression could be primarily a liquid-phase phenomenon, like in ESI.^37^ To investigate this, experiments were carried out with acetone and its deuterated analogue, D_6_-acetone. As isotopologues, their gas-phase reaction equilibrium constants with other reactive species are the same. Therefore, any observed differences would not be related to these equilibrium constants. Four experiments were conducted to examine the effect of higher acetone concentration levels in either the electrospray or the gas phase. In these experiments, the concentration of D_6_-acetone was kept constant in either the electrospray or the gas phase.

**Figure 3:**
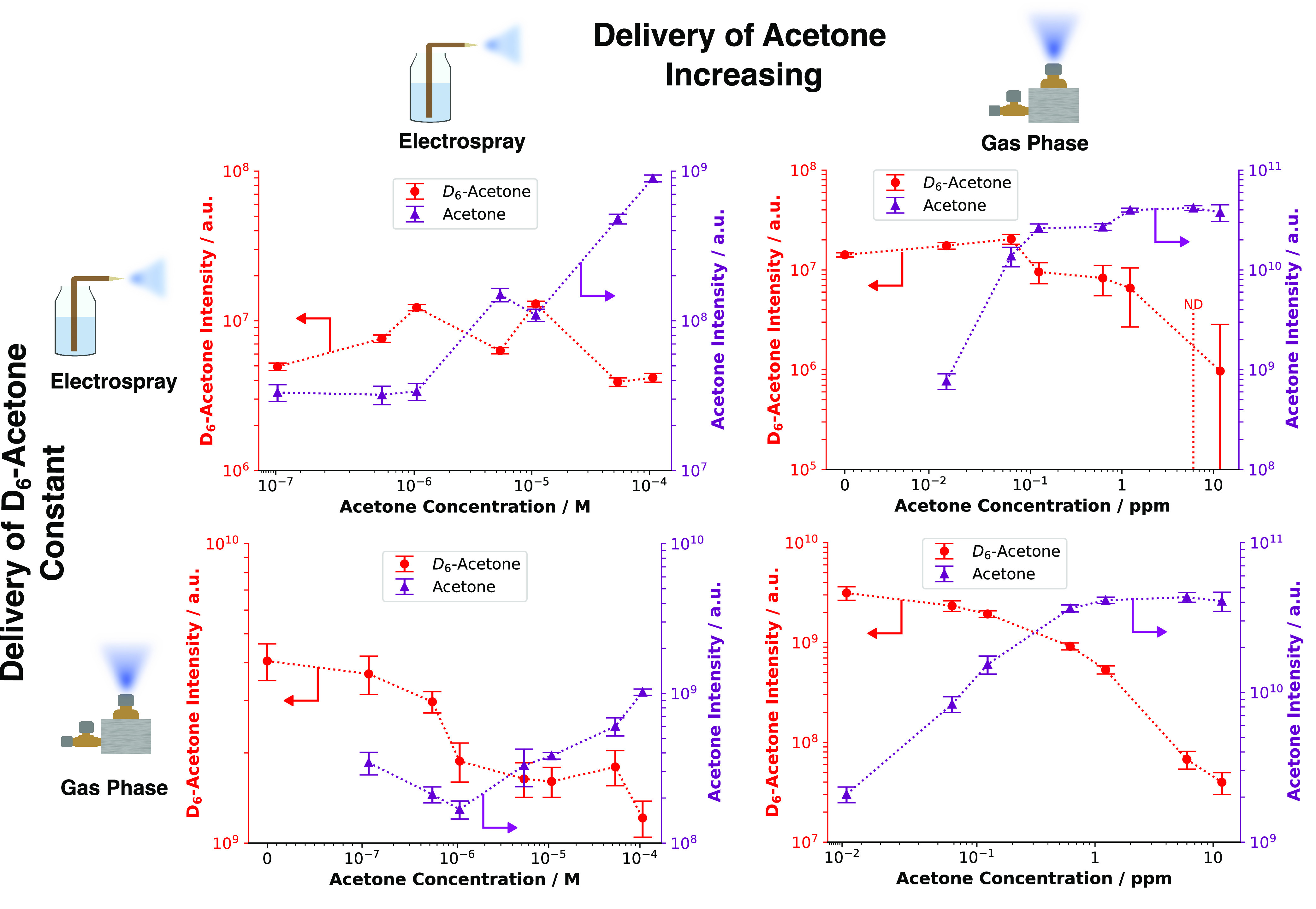
Signal intensities of acetone and D_6_-acetone as functions of increased acetone levels measured under dry conditions. “ND” indicates that the signal fell below the limit of detection of the mass spectrometer. Each row corresponds to a specific experiment, and the symbols at each end indicate how the compound was introduced into the ionization chamber: through either the electrospray or the evaporation chambers into the gas phase. The acetone concentration in the abscissa is plotted on a symlog scale, and the corresponding signal intensities on the ordinate are plotted on a log scale. Alteration of the acetone concentration in the electrospray appeared to have a less pronounced effect on D_6_-acetone compared to increasing the acetone concentration in the gas phase.

Figure 3 shows a clear difference between the increased levels of acetone in the electrospray and in the gas phase. In the upper panels of the figure, the concentration of acetone in the electrospray solution was increased. The signal intensity of D_6_-acetone stayed roughly the same in the electrospray and slightly decreased (by 1 order of magnitude) in the gas phase. In the lower concentration ranges (10^–7^ to 10^–6^ M), the signal of acetone did not increase with higher concentrations. This could indicate a change in the ionization efficiency of the spray through acetone.^38^ When the acetone concentration in the gas phase was increased, a more significant D_6_-acetone signal drop of roughly 2 orders of magnitude was observed compared to the initial signal strength. This occurred both in the electrospray and in the gas phase. At an acetone concentration of 1 ppm in the gas phase, a sharper decrease in the signal intensity of D_6_-acetone of about 1 order of magnitude was distinguishable when compared to the starting signal. Similar trends in the gas phase were observed for the same compounds (acetone and D_6_-acetone) under humid conditions as well (see Figure S1 in the Supporting Information). However, the drop in the signal intensity of D_6_-acetone was less pronounced under humid conditions (Figure S1) when compared with the measurements with higher acetone levels in the gas phase. The intensity difference between dry and humid conditions was a factor of 10. Additionally, a drop in intensity of D_6_-acetone under humid conditions (Figure S1) was observed for the gas-phase experiments only after acetone exceeded a concentration of 1 ppm, unlike under dry conditions (Figure 3). A slight drop in signal intensity was visible from the start (10^–2^ ppm). These observations correspond to the general signal decrease from dry to humid conditions for the same gas-phase concentration previously observed for short-chain fatty acids.^30^ A more intense analyte signal was detected under dry conditions when compared with humid conditions with the same stock solution and evaporation chamber settings. The data imply that an increase in acetone concentration in the gas phase affects the signal more severely than when acetone is present in the electrospray solution. With the reported experimental design, however, it is impossible to determine the contribution from a change in the droplet formation, which has been reported for some organic modifiers.^39,40^ Therefore, the actual concentration of the analyte released from charged droplets into the gas phase differs between the electrospray and gas phase.

**Figure 4:**
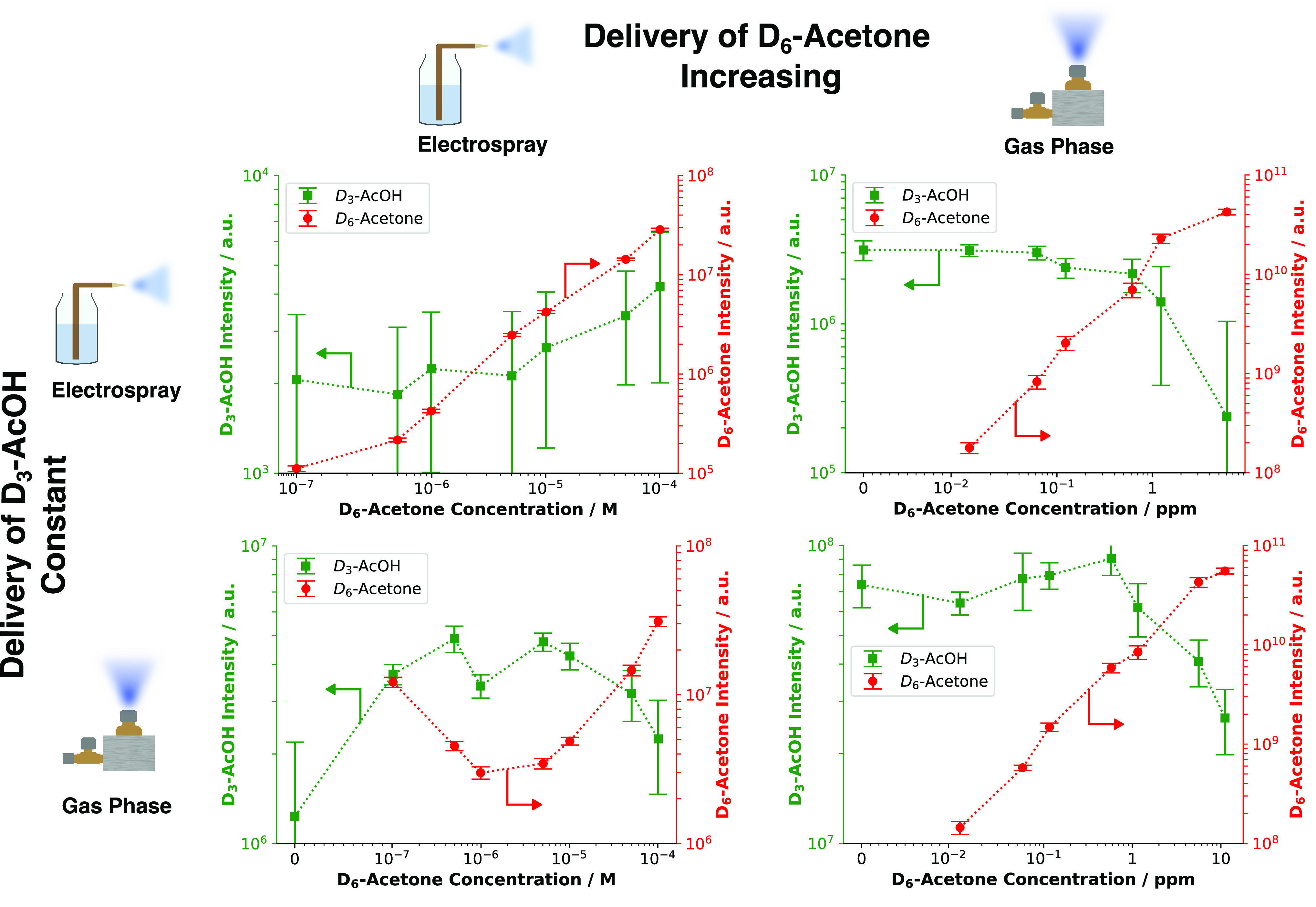
Signal intensities of D_6_-acetone and D_3_-AcOH as functions of increased D_6_-acetone concentration levels measured under humid conditions. Each row represents a different combination of electrospray and gas-phase measurements. The D_6_-acetone concentration is plotted on a symlog scale, which includes the control measurements recorded without any D_6_-acetone present. As D_6_-acetone levels increased in the gas phase, a decrease in the signal intensity of D_3_-AcOH in both the electrospray and the gas phase was observed.

To explore whether the findings obtained from the two isotopes of acetone could be expanded, the same experiment was conducted with D_6_-acetone against D_3_-AcOH. Both dry and humid conditions were applied, with the results of the humid measurements depicted in Figure 4.

The measurements conducted with D_6_-acetone added to the spray showed that, with one exception, the signal intensity changed by 1 order of magnitude under both humid (Figure 4) and dry (Figure S2) conditions. Similarly, measurements conducted with D_6_-acetone in the gas phase produced results similar to those obtained for increasing the concentration of acetone against D_6_-acetone. The signal of D_3_-AcOH in both the electrospray and the gas phase slightly decreased when D_6_-acetone levels were raised from 10 ppb to 1 ppm under dry conditions (Figure S2) or remained constant. However, after crossing a threshold of 1 ppm in humid conditions and 0.1 ppm in dry conditions, a steeper decrease in the signal intensity was observed. This difference between the acetone and D_3_-AcOH concentration in the onset of signal suppression indicates a contribution of gas-phase reactivity on top of the concentration.

This observation was supported by measurements with increased levels of D_3_-AcOH and constant levels of D_6_-acetone (see Figures S3 and S4). In the achievable concentration range of D_3_-AcOH, no clear trend regarding the decreased D_6_-acetone signal intensity was distinguishable. On the contrary, increased levels of D_3_-AcOH in the gas phase actually positively affected the signal strength of D_6_-acetone in the electrospray. This improvement in signal intensity resembled the effect of gas-phase modifiers, which alter and enhance the charging of analyte molecules.^41,42^ Similar trends were noticed with the acetone signal as in Figure 3, potentially suggesting that acetone changed the spray properties, which led to a change in the ionization efficiency of itself.

The observed trends among the compounds added in the electrospray and gas phases indicate that ion suppression in SESI does occur and is mainly dominated by gas-phase phenomena. No clear signal suppression was observed in the measured concentration range of additives in the electrospray solution. These findings expand on the results of King et al.^37^ for ESI. The gas phase can play a role with gas-phase ions suppressing the analyte in the electrospray solution. However, in LC-MS, such conditions are unlikely to arise, and the liquid-phase origins of ion suppression will be more prevalent.

With ion suppression present, the use of internal standards is necessary for more accurate quantification. As previously reported, internal standards added to the electrospray solution of the SESI source would be an option.^43^ Although this type of internal standard addition is an important method for quantification, it should be noted that relying solely on this method may not provide accurate results. This is because the signal intensities obtained from the gas phase and the electrospray exhibit variations that make them noncomparable, despite having the same concentration. One reason for this discrepancy is the expansion of the spray, which makes accurate concentration calculations impossible. However, an internal standard could serve as a probe for ion suppression because compounds in the electrospray react similarly to increased gas-phase levels, such as the analytes already present in the gas phase.

### 3.2 Signal Suppression Caused by Gas-Phase Compounds

**Figure 5:**
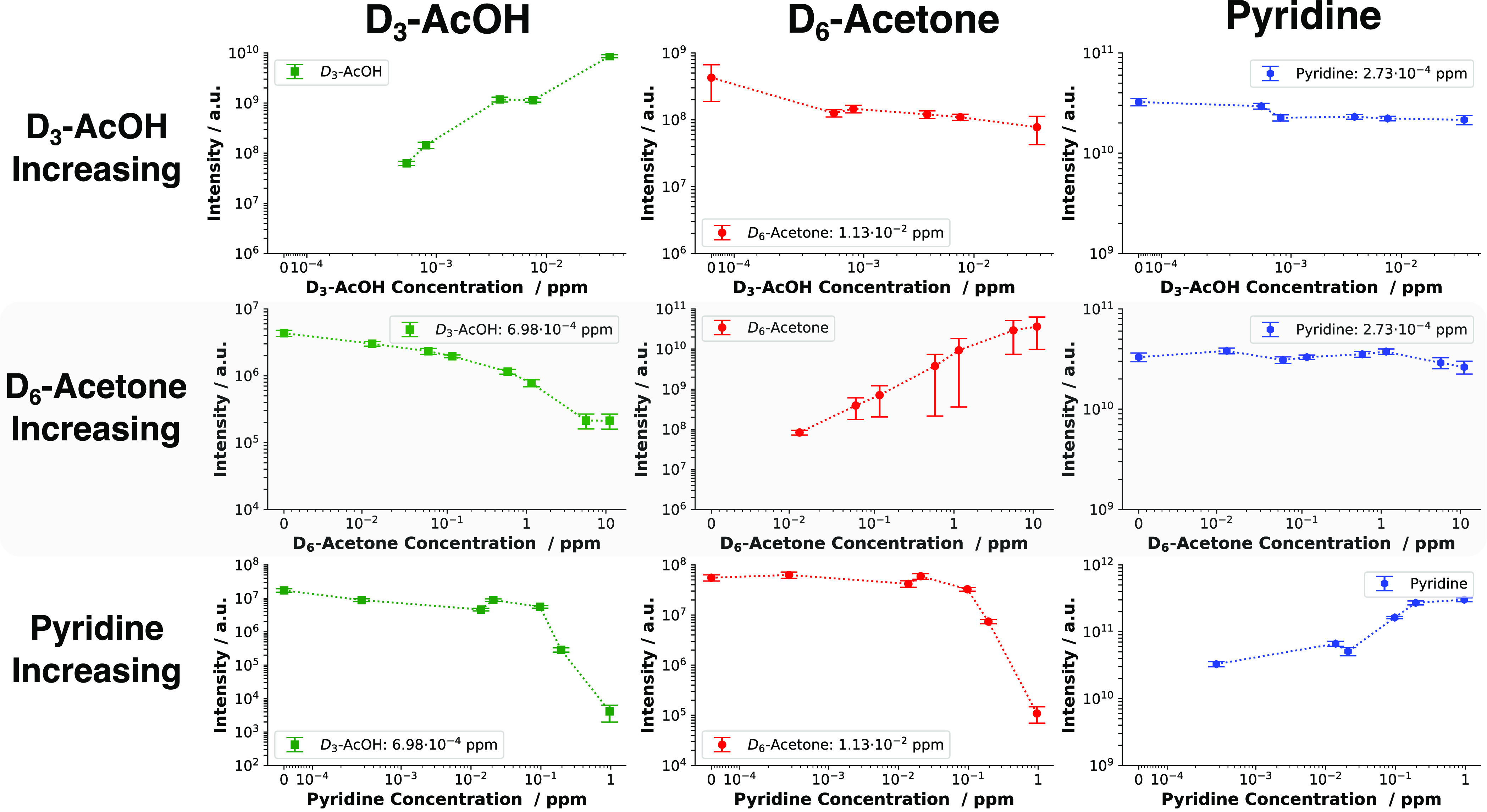
Signal intensities for D_3_-AcOH (green), D_6_-acetone (red), and pyridine (blue) as functions of the concentration of the compound with increasing concentration (indicated on the left) under humid conditions. The concentration values are displayed on a symlog scale, which includes the measurements recorded without the increased compound. The top row shows the signal intensities of the compounds tested when the concentration of D_3_-AcOH was increasing. In the middle row, the D6-acetone concentration was increasing while the other two remained constant. In the third row, the pyridine concentration was increasing while the D_3_-AcOH and D_6_-acetone concentrations remained constant. Elevated levels of D_3_-AcOH had a minimal impact on the signal of D_6_-acetone and did not at all affect pyridine. However, elevated levels of D_6_-acetone suppressed the signal of D_3_-AcOH while leaving pyridine unaffected. Elevated levels of pyridine suppressed the signals of both D_3_-AcOH and D_6_-acetone.

Additional experiments were conducted to investigate the impact of altering the gas-phase composition by increasing the concentration of one compound while keeping the other constant. Both compounds were introduced into the gas phase by evaporation chambers. Pyridine, a well-known component of exhaled breath, was added as an additional compound.^44^ A series of measurements (see Figures S5 and S6) were carried out with increasing D_6_-acetone levels against D_3_-AcOH and pyridine individually, along with increasing levels of D_3_-AcOH and pyridine against constant levels of D_6_-acetone. To enhance the sensitivity of the instrumentation further, the measurements were performed by cycling over the [M + H]^+^*m*/*z* ratios with a window of width 1. Therefore, the intrascan dynamic range was increased. Additionally, the three selected compounds were simultaneously measured, with one concentration increasing while the others remained constant. The resulting intensities are shown in Figures 5 (under humid conditions) and S7 (under dry conditions).

When the concentration of D_3_-AcOH in the gas phase was increased, a decrease in D_6_-acetone levels by approximately 1 order of magnitude (dry conditions, Figure S7) or half a decade (humid conditions, Figure 5) was observed. In both conditions, the signal of pyridine remained relatively stable. Conversely, when the concentration of D_6_-acetone was increased, the D_3_-AcOH signal dropped significantly, especially under dry conditions. Elevating the D_6_-acetone concentration in dry conditions by roughly 2 orders of magnitude led to a decrease in the D_3_-AcOH signal intensity by 4 orders of magnitude (Figure S7). The same increase in the D_6_-acetone concentration levels under humid conditions led to a D_3_-AcOH signal loss of less than 2 orders of magnitude. These results are in stark contrast to the relatively stable signal of pyridine: under humid conditions, no drop in signal intensity could be observed, and under dry conditions, only a small drop in the signal intensity was observed after D_6_-acetone exceeded a concentration of 1 ppm. The most potent effect on signal intensity was observed when the concentration of pyridine was increased. For both D_3_-AcOH and D_6_-acetone, a continuous loss of signal intensity was observed under both dry and humid conditions until a concentration of 10 ppb (dry) or 100 ppb (humid) was reached. Passing this threshold led to a more drastic loss of signal intensity for both D_3_-AcOH and D_6_-acetone, with both compounds exhibitng a loss of signal intensity of up to 4 orders of magnitude when pyridine levels reached 1 ppm.

As previously hypothesized, a potential explanation for the notable differences in susceptibility to ion suppression among the different analytes can be attributed to gas-phase acid–base chemistry.^6^ The gas-phase basicity, which represents the Gibbs free energy of the proton transfer reaction, is 752.8 for D_3_-AcOH, 782.1 for D_6_-acetone, and 898.1 kJ mol^–1^ for pyridine.^45^ The order of the basicities corresponds to the observed suppression effects for the individual compounds. Pyridine, with the highest basicity, suppresses both D_3_-AcOH and D_6_-acetone. In comparison, D_6_-acetone only affects D_3_-AcOH on a large scale, and D_3_-AcOH only affects D_6_-acetone in a minor way. If gas-phase basicity is the governing factor of ion suppression in SESI, this would mean that protonated analyte particles are deprotonated in the presence of a strong gas-phase base. Thus, fewer analyte ions would enter the mass spectrometer.

This rationale relies on proton transfer as the charging mechanism;^9^ however, it would not hold if the mechanism of SESI were to occur via ligand switching.^10^ In this scenario, suppression would occur based on the higher propensity of one analyte to bind charged water clusters compared to another. In this case, the equilibrium constant of the switching reaction would determine the severity of the suppression effect, assuming thermodynamic equilibrium has been established.

Under humid conditions, it was observed that the signal suppression effect was less severe, which corresponded to lower sensitivity under these conditions. Additionally, it is likely that the generated droplets caused some of the present analyte to be washed out. As a result, the concentration in the ionization chamber of the SESI source was reduced, leading to less ion suppression.

### 3.3 Estimation of Ion Suppression in Exhaled Breath

**Figure 6:**
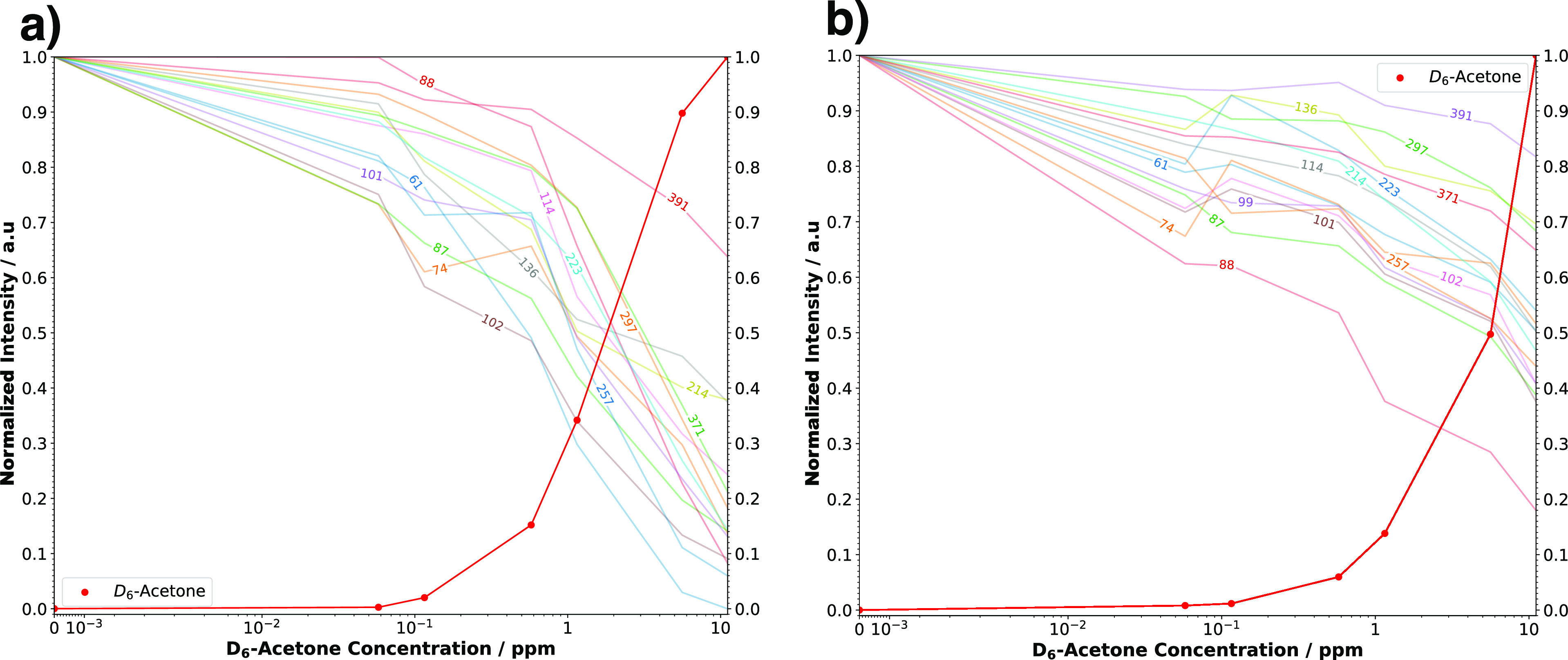
Normalized signal intensities of the features found in the gaseous phase of the collected breath condensate when D_6_-acetone levels were elevated under (a) dry and (b) humid conditions. Each feature is labeled with its nominal mass-to-charge ratio. Under both conditions, a signal intensity drop was observed as D_6_-acetone levels increased. Under dry conditions, this drop was more pronounced, with most features falling below 50*%* of the initial signal intensity at 1 ppm D_6_-acetone. Under humid conditions, most features lost at least 30*%* of their initial signal when the D_6_-acetone concentration reached 10 ppm.

To assess the impact of ion suppression on exhaled breath components, breath condensate was collected and used to generate a gas-phase sample by using an evaporation chamber, as described above in Section 2.2. While this condensate does not encompass all possible metabolites present in online exhaled breath samples, it serves as a good model system for studying actual exhaled breath. To maintain consistency, D_6_-acetone levels were incrementally increased to 10 ppm while ensuring a constant flow through the condensate-filled chambers. For each measurement, freshly thawed condensate was utilized to minimize the compound loss through evaporation. The detected features were categorized based on their overall trend into two classes: increasing features associated with D_6_-acetone, such as the hydrate or the dimer form, and decreasing features linked to compounds derived from the condensate. The normalized signal intensities of the decreasing features detected in the gas phase of the condensate under both dry and humid conditions are presented in Figure 6.

The features found in the gas phase of the condensate responded to increasing D_6_-acetone levels. The most significant effects were observed under dry conditions (Figure 6a), where features dropped to less than 50% of the initial intensity at a D_6_-acetone concentration of 1 ppm. Although less severe, significant signal loss for most features was also observed in measurements obtained with a humidified dilution flow (Figure 6b), with signal drops ranging from 20 to 80% when a D_6_-acetone concentration of 10 ppm was reached. It is impossible to know whether these more volatile compounds detected in the condensate are more likely to have low gas-phase basicity or to predict whether similar signal drops would occur for most exhaled breath components. Nevertheless, these results indicate the potential for ion suppression in exhaled breath analysis when certain compounds are present at sufficiently high concentrations.

Acetone is one of the more concentrated compounds found in the breath, with average concentrations ranging from 500 ppb to 1 ppm; it is also a compound with a considerable intrasubject variation, reaching concentrations up to 1000 ppm in diabetes patients.^29,46−48^ Excluding extreme levels of acetone in the breath, it is expected that a signal suppression of at least 10%, as seen in Figure 6, could occur with normal acetone levels. Various analytes with a lower gas-phase basicity than acetone could be affected, including carboxylic acids (e.g., short-chain fatty acids) and hydrocarbons (e.g., isoprene).

While acetone might be a crucial factor in ion suppression in SESI, other compounds such as ammonia may also contribute to ion suppression effects in exhaled breath due to their relatively high concentrations. Ammonia could not be detected with the mass spectrometer that was used in this study, but it has similar concentration levels in breath compared to acetone^46,49^ and has a gas-phase basicity of 819.0 kJ mol^–1^.^45^ Therefore, it is likely that ammonia also suppresses the signal in SESI-MS, necessitating consideration in the analysis.

Consequently, two concerns arise when conducting SESI-MS experiments, regardless of the mass spectrometer used. First, the interpretation of metabolic trends may be influenced by changing acetone or ammonia levels, thus potentially overshadowing the true biological variation in an organism. Therefore, individual trends should be compared to acetone levels to avoid the misinterpretation of metabolic data due to acetone variation. Second, for quantification purposes, external calibration of the SESI-MS system may not be feasible for exhaled breath if the analyte is suppressed by acetone. Conducting a suppression experiment with increased acetone concentrations is necessary to determine whether a compound is suitable for external calibration. Additionally, acetone could decrease the signal of a feature below the limit of detection of the mass spectrometer, leading to the potential omission of important biomarkers in metabolomic studies.

### 3.4 Approaches to Mitigate Ion Suppression

If quantitative analysis is not required or if the sample has low complexity, ion suppression can most likely be disregarded. Monitoring amines, for example, generally requires no additional measures, as they are less susceptible to suppression. However, in order to mitigate ion suppression effects for susceptible compounds in SESI-MS, various approaches could be employed. These would aim to improve the ionization efficiency of analytes and minimize the interference caused by competing ions or sample matrix components. The choice of strategy depends on the type of sample that is being analyzed and the chemical nature of the analytes of interest.

Operating under humid conditions is an option to consider for experiments involving a carrier gas flow. The data presented here indicate that ion suppression is less significant under humid conditions; i.e., humidifying the carrier gas flow can be a viable approach for decreasing ion suppression by approximately 30% (Figure 6). However, it is essential to note that humidification may reduce the sensitivity for specific compound classes.^30^ Therefore, understanding how the humidity level of the carrier gas affects the analysis performance is crucial.

Another approach to mitigate the impact of suppression is to dilute the sample with an additional gas flow, as demonstrated in Figure 6. Dilution can effectively reduce suppression, but its success depends on the concentration of the suppressing compounds and the chemistry of the suppressed compounds. In exhaled breath analysis, this would be influenced by highly abundant breath components, such as acetone. If the acetone concentration exceeds 1 ppm, a dilution factor of at least 100 would be necessary to reduce suppression below a 20% relative signal loss (Figure 6). However, it is important to note that this will inevitably result in a trade-off with sensitivity. Therefore, a balance needs to be struck between minimizing suppression and maintaining the sensitivity of the measurements.

The use of isotopically labeled internal standards can be helpful in correcting ion suppression effects. By incorporating labeled analogues of the analytes of interest, it is possible to measure and compensate for variations in ionization efficiency. Labeled internal standards behave like the target analyte during ionization, allowing for a more accurate quantification and a correction of the ion suppression effects. Additionally, by implementing selective ionization techniques (e.g., carefully selecting the electrospray solution), the ionization of target analytes could be enhanced by minimizing the interference from other compounds present in the matrix. Optimizing the composition of the electrospray solution would improve the ionization efficiency and reduce the impact of ion suppression. Isolating and removing unwanted matrix components from the sample (e.g., by selective filtering in the sampling line) may also reduce the impact of ion suppression. This selective filtering process could help reduce the impact of ion suppression by minimizing the presence of interfering substances in the sample that could affect the ionization efficiency of the target analytes. These strategies could contribute to the more accurate and reliable quantification of metabolites in breath metabolomics.

## 4 Conclusions

To comprehensively characterize ion suppression in SESI for quantitative analysis, a gas standard generation system based on evaporation was employed. The investigation focused on the impact of increased concentrations of selected compounds in both the electrospray and the gas phase. The results revealed that elevated gas-phase concentrations of the suppressing compound were primarily responsible for signal reductions.

A comparative analysis of D_3_-AcOH, D_6_-acetone, and pyridine showed that D_3_-AcOH did not suppress the signals of the other compounds, while D_6_-acetone significantly affected the signal of D_3_-AcOH and minimally affected pyridine. Pyridine exhibited the strongest suppressive effect, decreasing the signal intensities of both D_3_-AcOH and D_6_-acetone. Furthermore, higher concentrations of the suppressing compound were required under humid conditions to observe the intensity drop compared to dry conditions.

The ion suppression effect of acetone on exhaled breath was assessed using D_6_-acetone and exhaled breath condensate. When the gas-phase levels of D_6_-acetone were increased to 10 ppm, the intensity of volatile components in the condensate decreased by approximately 50%. These findings confirm the presence of a significant presence of ion suppression effects in SESI. Extrapolating from the data, compounds with lower gas-phase basicity are expected to be more strongly suppressed, making their quantification challenging through external calibration.

It should be noted that the experimental approach employed here did not account for space charge effects occurring between the mass spectrometer inlet and quadrupole. Therefore, the observed suppression effects may overlap with some Coulombic interactions between ions.

Further experiments are necessary to gain a deeper understanding of the susceptibility of selected compound classes to ion suppression. It is also crucial to develop additional mitigation strategies to enhance the data quality and improve the interpretation of the results.

## Data Availability

The original data underlying this study are openly available in a curated data archive at ETH Zürich (https://www.research-collection.ethz.ch) under DOI: 10.3929/ethz-b-000614438.

## Supporting Information Available

Humid condition results for D_6_-acetone versus acetone, dry condition results for D_3_-AcOH versus D_6_-acetone, results of increasing D_3_-AcOH against D_6_-acetone under dry and humid conditions, results of increasing individual compounds against each other, and the three compound results under dry conditions (PDF)
