# Supplementary material for: Elucidating the Role of Ion Suppression in Secondary Electrospray Ionization
Source: J Am Soc Mass Spectrom. 2023 Oct 16;34(11):2498–507. doi: 10.1021/jasms.3c00219 (PMC10623576; doi:10.1021/jasms.3c00219)
Supplement: Supplementary file 1 — js3c00219_si_001.pdf [file js3c00219_si_001.pdf]

SI to "Elucidating the Role of Ion Suppression in Secondary Electrospray Ionization"

Cedric Wüthrich[1], Stamatios Giannoukos[1]*, Renato Zenobi[1]*

(1) Department of Chemistry and Applied Biosciences, ETHZ, 8093, Zurich, Switzerland

*Correspondance: Stamatios Giannoukos (stamatios.giannoukos@org.chem.ethz.ch), Renato Zenobi (zenobi@org.chem.ethz.ch)

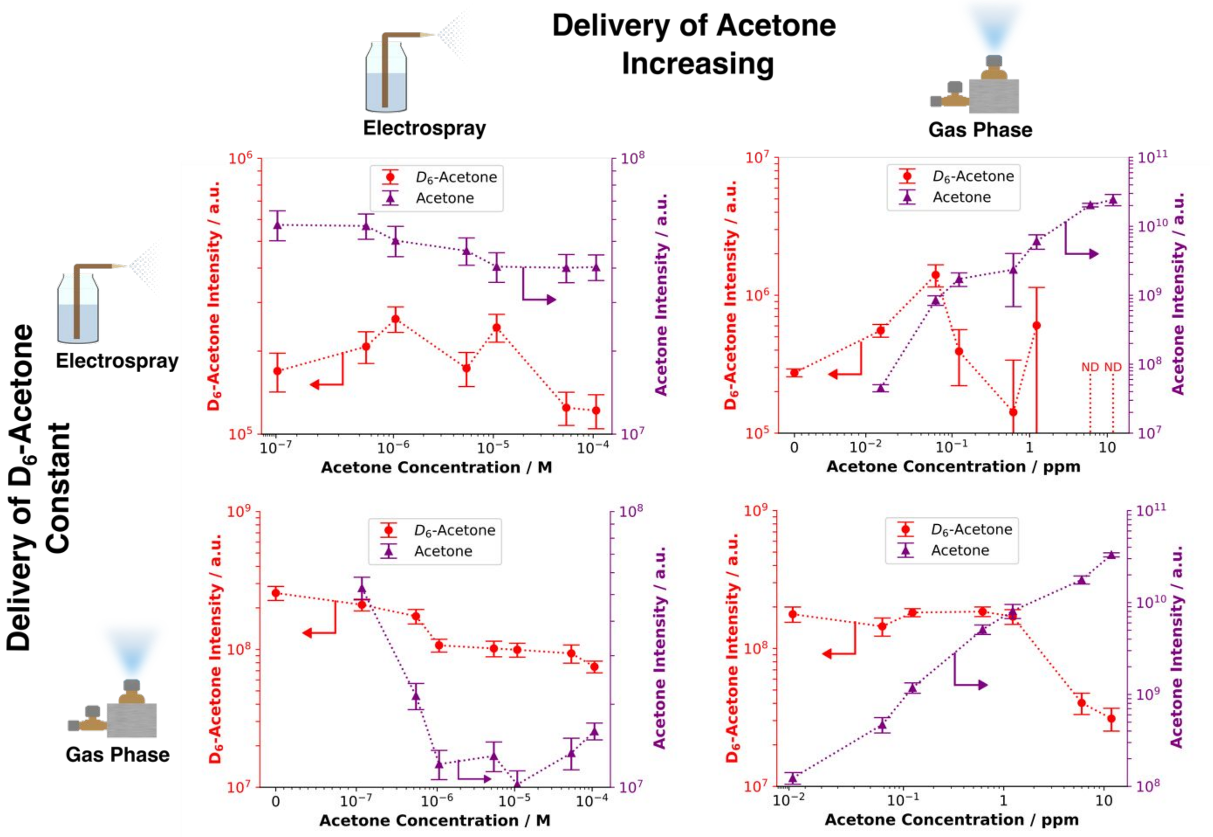

Figure S1. Signal intensities of acetone and D_6-acetone as a function of increasing acetone concentration measured under humid conditions. In the top row (left side), both compounds were added to the electrospray, and no noticeable shift in signal intensity occurred. Conversely, at the top row (right side), the acetone concentration in the gas phase was increased while D_6-acetone remained constant. "ND" indicates measurements where no signal was detected. The bottom panel (left side) shows the intensities when acetone was increased in the electrospray while D_6-acetone levels remained constant in the gas phase. The results in the bottom right sub-figure depict the signal intensities when both compounds were in the gas phase, achieved through the evaporation chamber.

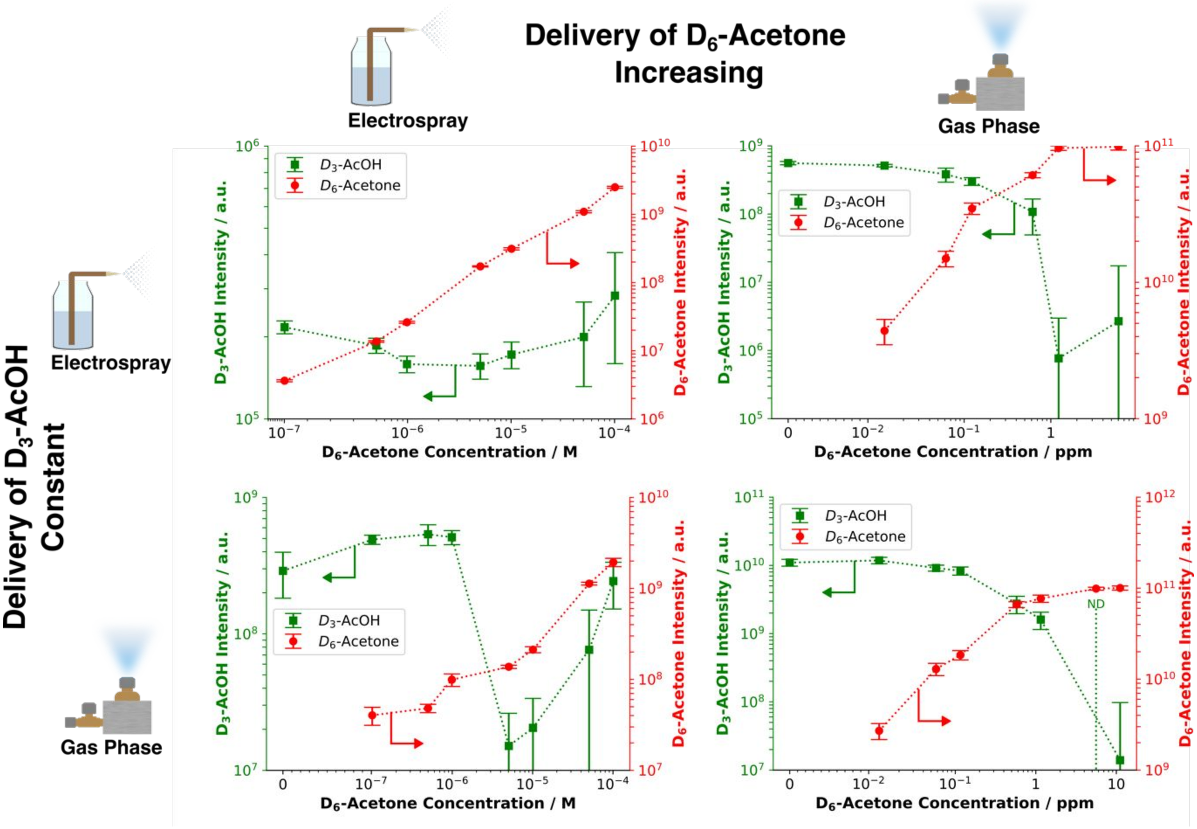

Figure S2. Signal intensity of D_6-acetone and D_3-AcOH as the concentration of D_6-acetone increases under dry conditions. The top-left subfigure shows the results when both compounds were present in the electrospray. In the top-right and bottom-left sub-figures, the signal intensities are shown when one compound was in the electrospray while the other was in the gas phase. The bottom-right sub-figure shows the results of when both compounds were present in the gas phase. "ND" indicates that the signal was not detected. D_6-acetone had the most significant impact on suppressing the signal of D_3-AcOH, whether it was in the electrospray or the gas phase.

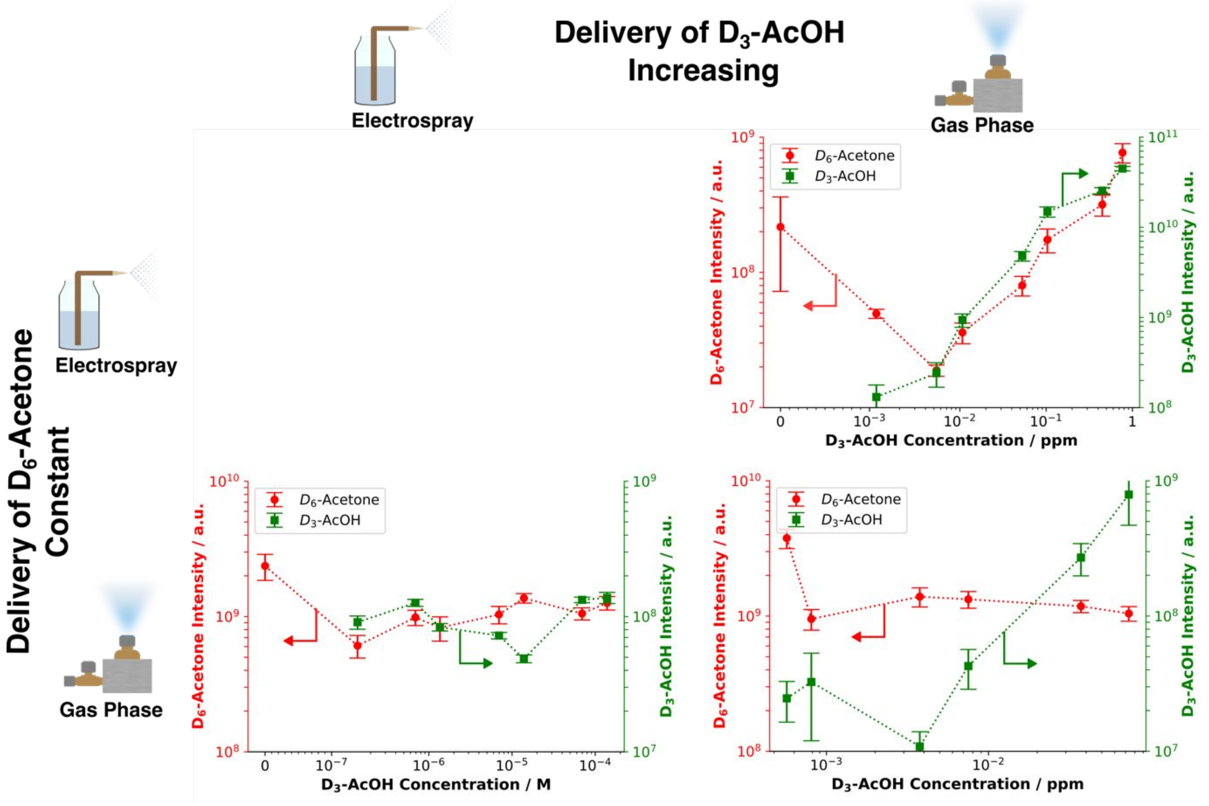

Figure S3. Signal intensities of D_3-AcOH and D_6-acetone as the concentration of D_3-AcOH was increased under dry conditions. In the bottom-left sub-figure, the signal intensities are shown when the concentration of D_3-AcOH was increasing in the electrospray while the gas phase concentration of D_6-acetone remained constant. The top-left sub-figure showed the reverse, with D_3-AcOH having been increased in the gas phase. Interestingly, when D_3-AcOH reached a sufficiently high concentration, the signal of D_6-acetone was boosted. However, as shown in the bottom-right sub-figure, this phenomenon was not observed when both compounds were in the gas phase.

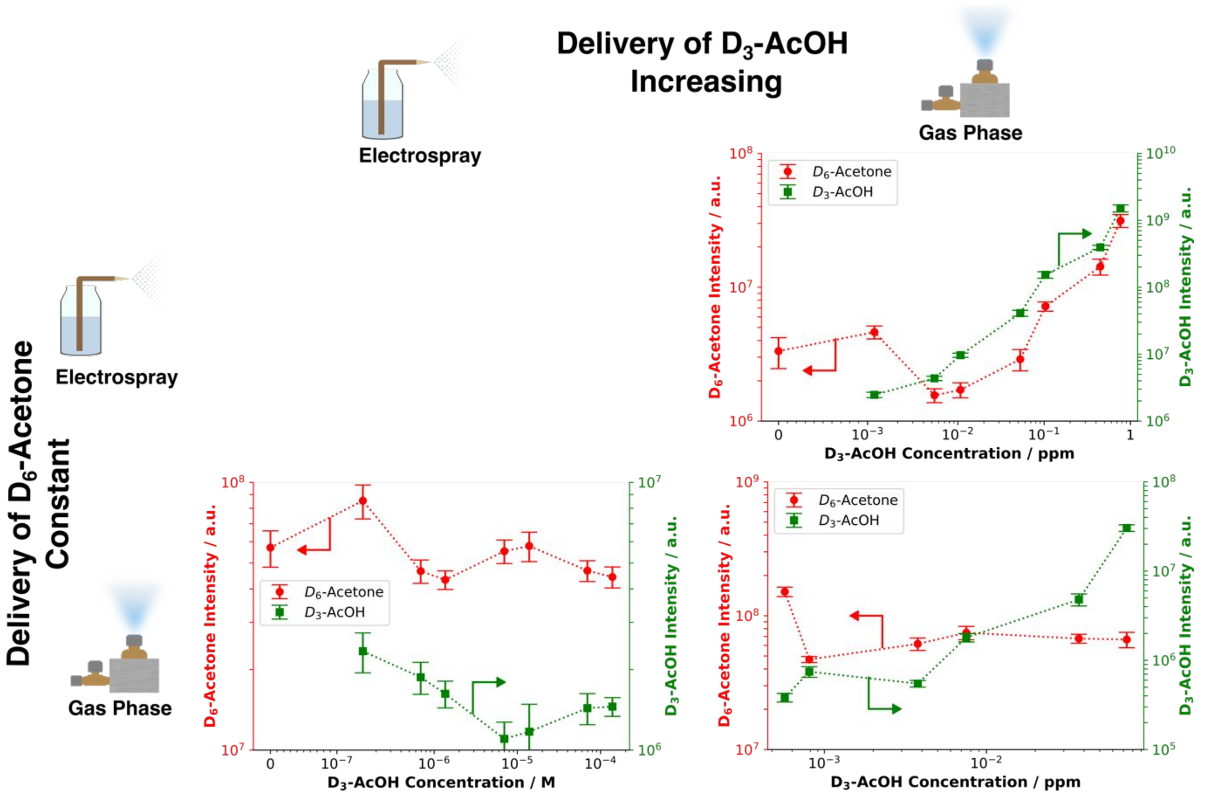

Figure S4. Signal of D_3-AcOH and D_6-acetone when the concentration of D_3-AcOH was increased under humid conditions. In the bottom-right sub-figure, the levels of D_3-AcOH in the electrospray were raised while the concentration of D_6-acetone remained constant in the gas phase. The top-right sub-figure demonstrated the opposite scenario, where the concentration of D_3-AcOH was increased in the gas phase. The bottom-right subfigure displays the signals when both compounds were introduced as gases into the ionization chamber.

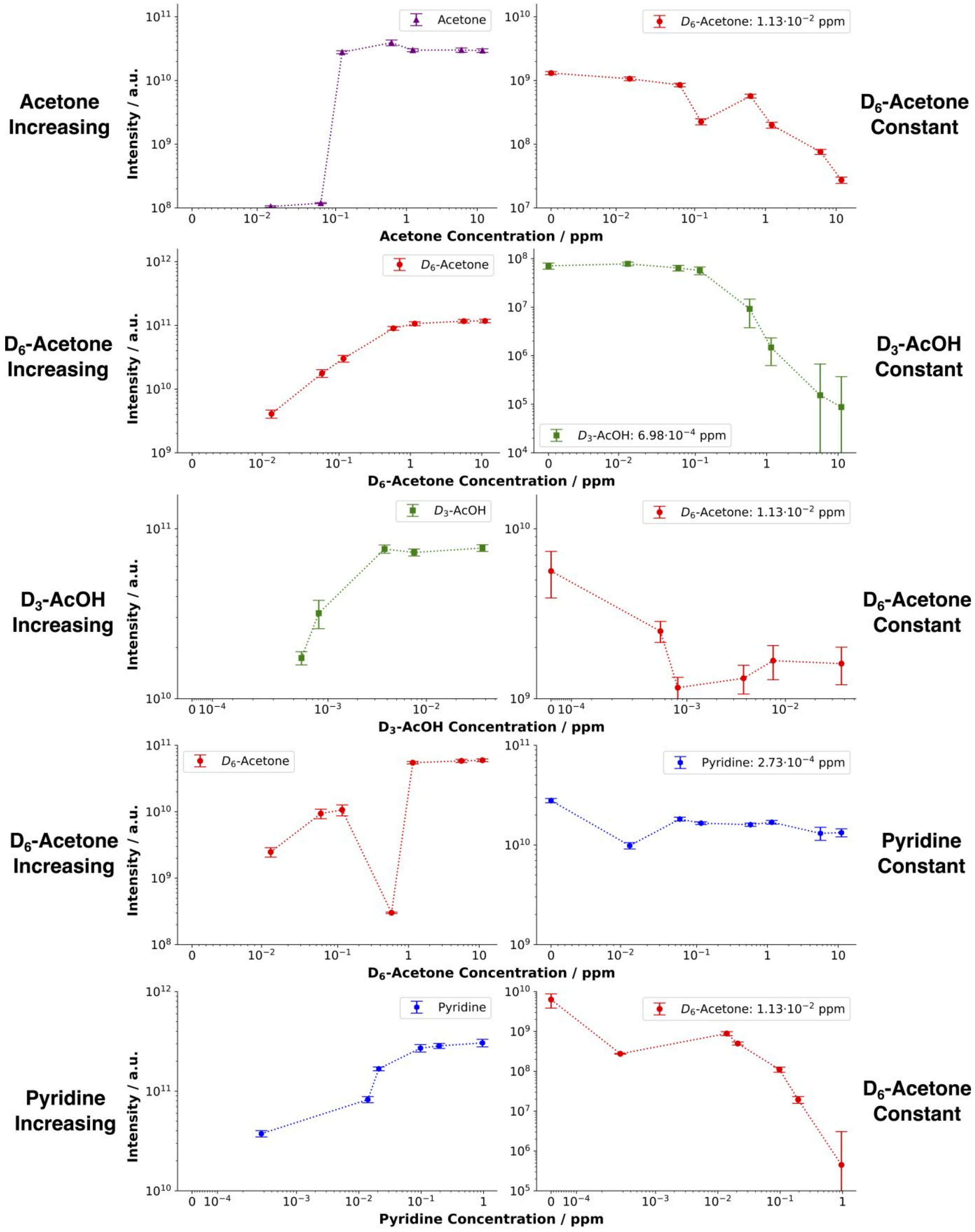

Figure S5. Signal intensities of several selected compounds introduced to the ionization chamber through evaporation chambers under dry conditions. The intensities are shown as a function of the concentration of the specific compound being increased.

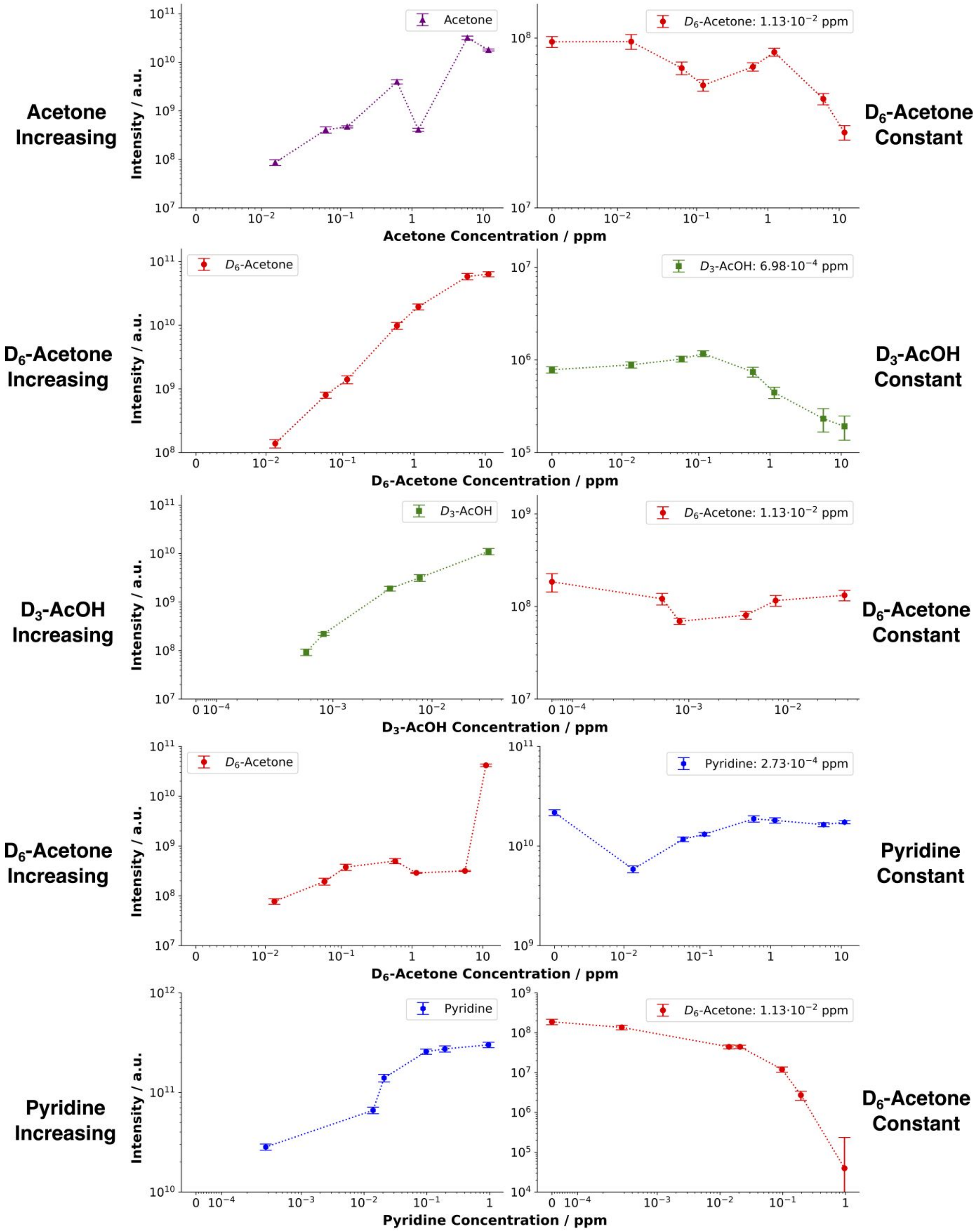

Figure S6. Signal intensities of several selected compounds introduced to the ionization chamber through evaporation chambers under humid conditions. The intensities are presented in relation to the concentration of the respective compound being increased.

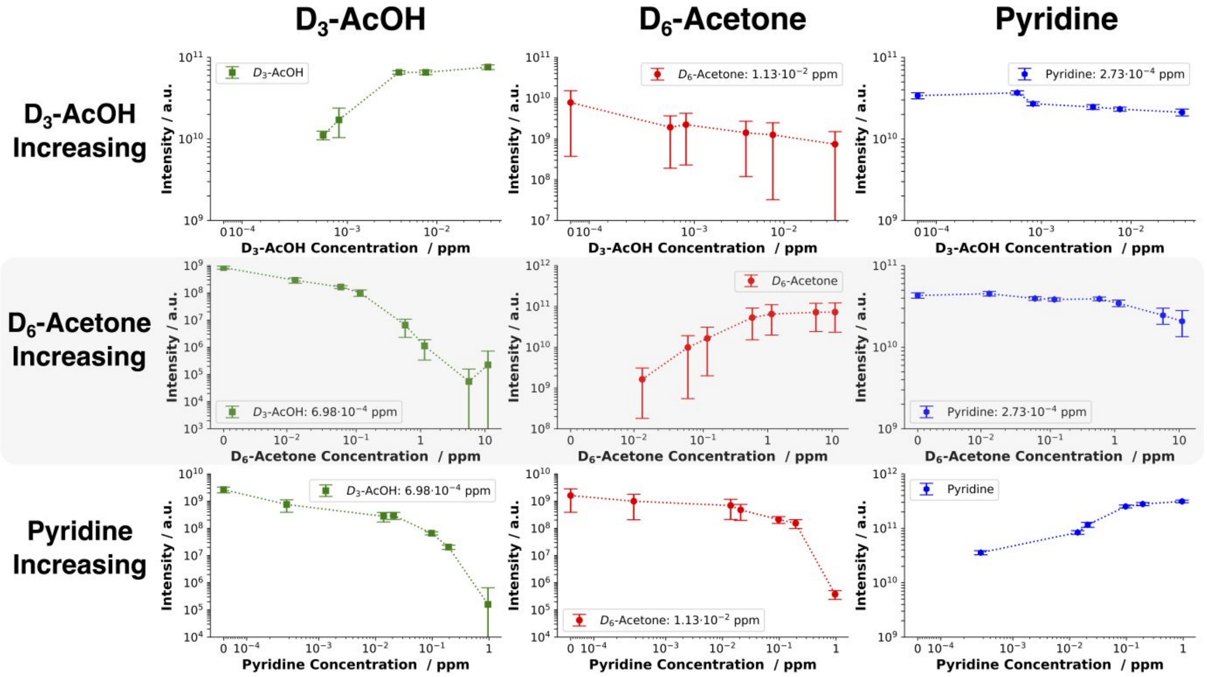

Figure S7. Signal intensities of D_3-AcOH, D_6-acetone and pyridine as a function of the increased compounds. These measurements were recorded after generating gas standards of the individual compounds through evaporation chambers. The first row shows the results when D_3-AcOH was increased, the second row shows the results for D_6-acetone, and the third row presents the results when pyridine was increased.
